# An evolutionary roadmap to the microtubule-associated protein MAP Tau

**DOI:** 10.1186/s12864-016-2590-9

**Published:** 2016-03-31

**Authors:** Frederik Sündermann, Maria-Pilar Fernandez, Reginald O. Morgan

**Affiliations:** Department of Neurobiology, University of Osnabrück, Osnabrück, Germany; Department of Biochemistry and Molecular Biology, Edificio Santiago Gascon 4.3, Faculty of Medicine, University of Oviedo, 33006 Oviedo, Spain

**Keywords:** Microtubule associated protein Tau (MAPT protein, *MAPT* gene), Microtubule binding domain, Gene phylogeny, Molecular evolution, Profile hidden Markov models, Saitohin (STH), Domain architecture, Structure-function prediction

## Abstract

**Background:**

The microtubule associated protein Tau (MAPT) promotes assembly and interaction of microtubules with the cytoskeleton, impinging on axonal transport and synaptic plasticity. Its neuronal expression and intrinsic disorder implicate it in some 30 tauopathies such as Alzheimer’s disease and frontotemporal dementia. These pathophysiological studies have yet to be complemented by computational analyses of its molecular evolution and structural models of all its functional domains to explain the molecular basis for its conservation profile, its site-specific interactions and the propensity to conformational disorder and aggregate formation.

**Results:**

We systematically annotated public sequence data to reconstruct unspliced MAPT, MAP2 and MAP4 transcripts spanning all represented genomes. Bayesian and maximum likelihood phylogenetic analyses, genetic linkage maps and domain architectures distinguished a nonvertebrate outgroup from the emergence of *MAP4* and its subsequent ancestral duplication to *MAP2* and *MAPT*. These events were coupled to other linked genes such as *KANSL1L* and *KANSL* and may thus be consequent to large-scale chromosomal duplications originating in the extant vertebrate genomes of hagfish and lamprey. Profile hidden Markov models (pHMMs), clustered subalignments and 3D structural predictions defined potential interaction motifs and specificity determining sites to reveal distinct signatures between the four homologous microtubule binding domains and independent divergence of the amino terminus.

**Conclusion:**

These analyses clarified ambiguities of MAPT nomenclature, defined the order, timing and pattern of its molecular evolution and identified key residues and motifs relevant to its protein interaction properties and pathogenic role. Additional unexpected findings included the expansion of cysteine-containing, microtubule binding domains of MAPT in cold adapted Antarctic icefish and the emergence of a novel multiexonic saitohin (*STH*) gene from repetitive elements in *MAPT* intron 11 of certain primate genomes.

**Electronic supplementary material:**

The online version of this article (doi:10.1186/s12864-016-2590-9) contains supplementary material, which is available to authorized users.

## Background

The microtubule associated protein Tau (MAPT) belongs to a family of homologous proteins, including MAP2 and MAP4, with 3 or 4 basic microtubule binding domains (MTBDs) in their carboxy terminal regions. The amino terminus may also interact with microtubules but precise functional interactions are poorly understood [1]. The 3 members of the MAPT/MAP2/MAP4 family are expressed as multiple splice variants, some of which contain different numbers of MTBDs [2]. MAPT and MAP2 are expressed mainly in neurons where they show a characteristic subcellular compartmentalization, with MAP2 being somatodendritic, MAPT predominantly present in the axon and MAP4 a major non-neuronal MAP.

MAPT is a natively disordered protein which can adopt dynamic conformations [3]. Intrinsically disordered proteins account for a substantial proportion of the proteome and many of them are promiscuous binders that undergo a partial transition to a more ordered state in which they interact stably with various partners and frequently function as molecular hubs in protein interaction networks [4–6]. Primary and posttranslational modifications of MAPT can compromise its physiological role in microtubule assembly and in mediating other cellular functions [7–10]. They might also contribute to aggregate formation in central neurons that are pathognomonic for Alzheimer’s disease and other “tauopathies” and could create MAPT species with toxic properties [11]. The regulation of MAPT expression and epigenetic contributions to it remain to be fully characterized and complex alternative splicing patterns depend on species, tissue and condition [12, 13]. The dynamic internal and external interactions of MAPT are influenced by primary sequence variation, post-translational modifications and polarized charge distribution that determine its site-specific properties responsible for physiological function and neuropathogenic effects [7, 12, 14].

The determination of MAPT functional organization has been hampered by two obstacles. First, only fragments of crystallographic structural information are available for MAPT due to its property as a natively disordered protein [6]. Second, mice lacking MAPT do not have major phenotypic changes indicating that functional redundancies may exist between MAPs. However, genetic data have provided evidence that MAPT is required for the normal development of the human brain since deletions at the locus are associated with severe developmental problems in children [15, 16].

The evolutionary history, key structural motifs and protein properties responsible for functions of the three paralogous vertebrate subfamilies MAPT, MAP2 and MAP4 comprise the main focus of this study. Previous studies have not yet resolved the full species distribution nor duplication order of MAP proteins [2, 17]. The binding of intrinsically disordered proteins to cellular structures and molecular partners is difficult to predict but can influence folding properties and protein turnover [18]; hence there is a need to compile full-length proteins from a broad range of species to obtain a reliable “roadmap” of all potential interaction motifs and domains. Such a comprehensive view overcomes the limitation of studying partial isoforms and highlights all features potentially responsible for the full functionality of MAPT. We have therefore undertaken a molecular evolution study of the MAPT/MAP2/MAP4 gene superfamily and identified significantly conserved features and patterns of divergence in MAPT that are likely to be responsible for some of the observed protein properties and cellular interactions. This approach has been successfully applied to reveal insight into other protein families [19–22].

## Results

### Exon organization and transcript splicing of human MAPs with tau-like microtubule binding domains

**Fig. 1:**
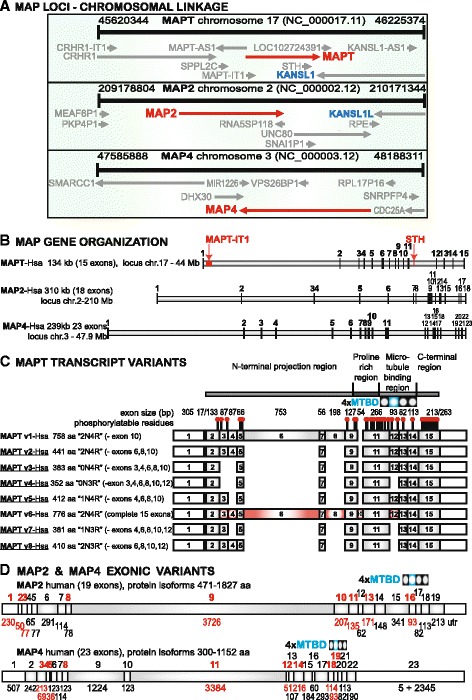
Gene organization and transcript variants of human MAPs with tau-like microtubule binding domains. **a** Chromosomal loci and genetic linkage maps of human *MAPT*, *MAP2* and *MAP4*, including *MAPT-IT1* and *STH* within the *MAPT* gene. There is also evidence that segmental chromosome duplications 17 ↔ 2 and 17 ↔ 3 formed the paralogous gene pairs *MAPT-KANSL1* and *MAP2-KANSL1L* (see text). **b** Official gene names and sizes identify the graphic outlines of their respective exon distributions. *MAPT* intron 1 contains *MAPT-IT1* (intronic transcript 1, long non-coding RNA), intron 11 contains the saitohin gene (*STH*) encoding a single open reading frame and peptide, while numerous remaining non-coding regulatory RNAs and repetitive elements in other introns are not annotated here. **c** Alternatively spliced transcripts of 8 human MAPT isoforms are identified by formal and familiar terminology showing size distributions of untranslated and coding (grey-filled) exons. The descriptive summary of protein products corresponds to different alternatively spliced exons produced by skipping of one or more exons 3, 4, 6, 8, 10 and 12 in different cell types and conditions; note that a previous, non-standard nomenclature restricted to the 6 brain isoforms started numbering at 1 for the first “coding” exon 2 while exon 4A was designated for exon 6 leading to an apparent maximum exon number of 13 instead of the true 15 [12]. The 4 MTBDs are marked at the top, the second one in color to denote the possible splicing out of exon 12. Known and predicted phosphorylation sites are identified by the ball and stick symbol above MAPT isoform 1. Note that the underlined 6 protein isoforms (v2, v3, v4, v5, v7, v8) are expressed in the central nervous system. Experimental evidence for the expression of exon 10 in humans is still lacking (NCBI BLASTN human RefSeq transcripts). A schematic representation showing the functional organization of tau is displayed on top. **d** Human *MAP2* and *MAP4* coding (grey-filled), non-coding and alternatively spliced exons (red numbers) are shown to characterize protein isoforms and localize MAP domains, including the exon splicing affecting the second MTBD

The current state of knowledge about gene, transcript and protein structures of human microtubule-associated proteins (MAPs) is summarized in Fig. 1 as a reference basis for our manual annotation of all exons in novel homologs from the 7 taxonomic classes of vertebrates. The most significant, canonical feature consists of 3–4 tandemly repeated microtubule binding domains (MTBDs) rich in basic residues within the carboxy terminal and principally required for the nucleation and elongation of microtubules and the dynamic interaction with the microtubule surface [23, 24]. Information was extracted from the latest genomic contigs and official nomenclature at the National Center for Biotechnology Information [25]. Two genes internal to *MAPT* are designated *MAPT-IT1* (long non-coding RNA intron 1 transcript) and the saitohin (*STH*) single exon open-reading frame in *MAPT* intron 11 that is scrutinized here later. Numerous other genomic repetitive elements, regulatory RNAs and single nucleotide polymorphisms also relevant to MAPT function are treated by example below. The MAPT locus is known to be susceptible to microduplications and microdeletions, and an ancestral inverted H2 haplotype predominant in European Causasians contrasts with the direct-oriented H1 haplotype more closely associated with neurodegenerative diseases [26].

An observation that complements later phylogenetic analysis of MAP evolution is that the *KANSL1* gene (subunit of histone acetyltransferase activity responsible for epigenetic modification of chromatin) adjacent to *MAPT* on chromosome 17q21.31 has a homolog *KANSL1L* near *MAP2* on chromosome 2q34 and a third paralog *KANSL3* on chromosome 2q11.2. *KANSL1* protein is an evolutionarily conserved regulator of the chromatin modifier KAT8, which influences gene expression through histone H4 lysine 16 (H4K16) acetylation [27]. These linked genes form a recurrent deletion that encompasses five known protein-coding genes, *CRHR1, SPPL2C, MAPT, STH* and *KANSL1*, in addition to two putative genes, *MGC57346* and *C17orf69* [26]. The *HOX*-bearing chromosomes (2, 7, 12, 17) are known to contain paralogon groups, analogous to other human chromosomes 1/6/9/19, 4/5/8/10 and 1/2/8/10, formed during the hypothesized two rounds of whole/segmental genome duplications at the inception of vertebrates [28, 29]. The possibility that *MAPT* and *KANSL1* may be functionally linked is consistent with the finding that these genes (like *MAP2* and *KANSL1L*) have been in genetic linkage since separation of the earliest extant vertebrates, based on our analysis of lamprey and hagfish contigs, and that they comprise known paralogon groups between human chromosomes 2 and 17 [28] and update of Human Chromosomal Paralogons [30]. Thus, the 17q21.31 linkage group (*MAPT, KANSL1, ADAM11, MYL4* = myosin light chain 1) forms a 4 Mb paralogon group corresponding to genes at 2q11.2 (*MAP2, KANSL1L, ADAM23, MYL1*) and 3p21 (*MAP4, MYL3*). The MAP paralogs *MAPT* and *MAP2* also show a species distribution and phylogeny comparable to *KANSL1* and *KANSL1L* [31], text below.

The distribution and sizes of exons are presented in detail for *MAPT*, *MAP2* and *MAP4* (Fig. 1) to show the congruity of the C-terminal 5 exons and to identify (in red) all potential alternatively spliced exons. The official and familiar nomenclature is shown for 8 human MAPT isoforms encoded by transcript variants modified by the exon deletions indicated. The underlined variants (v2, v3, v4, v5, v7, v8) encode isoforms that are expressed in the central nervous system and are subject to extensive post-translational modification and prone to aggregation as a causative or contributing factor in various tauopathies [10]. Note that some earlier publications modified MAPT nomenclature by designating the first untranslated exon as “-1” and renaming alternatively spliced exon 6 as exon 4A to enumerate 13 instead of the 15 consecutive exons [12]. The paralogous genes *MAP2* and *MAP4* have distinct tissue expression patterns in the nervous system and non-neural tissues, respectively, but contain homologous MTBD and are subject to comparable variation in alternative splicing. It should be emphasized that the patterns of alternative splicing in the MAPs are peculiar to individual tissues, species and conditions in a highly regulated process. Taken together the data indicate that *MAPT* and *MAP2* are genetically linked to larger paralogon groups.

### Phylogenetic analysis of proteins with Tau-like MTBDs

The relatively low sequence identity/similarity of MAP amino terminal projections and central domains could benefit from computational phylogenetic and statistical HMM analyses of a more extensive species range to validate conserved regions of functional importance and sites responsible for functional divergence. The need for accurate, extensive alignments of true homologs to resolve phylogenetic analyses and yield informative molecular profiles of functional regions implicitly demands full-length protein sequences based on all exons from the broadest range of species possible. Intrinsically disordered proteins commonly have multiple interaction partners, exemplified by the >16 high confidence, experimentally determined binding partners for MAPT in the STRING protein interaction database [32]. While attention has naturally focused on the C-terminal MTBD [18], the amino terminal projection domain may also undergo entropic repulsion [33] or electrostatic attraction (see later Results, Fig. 4d) or more specific, ligand-based interactions dependent on conformational changes yet to be defined. The aim of tracing the evolutionary origins and relationships between MAPs and classifying the most distant homologs in early-diverging vertebrates thus required primary reconstruction and annotation of many novel MAP homologs, followed by the application of pHMM models for individual missing exons and full-length transcripts to verify new members. Homologs were culled by PSI-BLAST and JACKHMMER searches of public sequence databases at the National Center for Biotechnology Information (NCBI) and UniProtKB, while those not yet catalogued in public databases were deduced and completed from recently sequenced genomes of mammals, reptiles (turtles, lizards and crocodiles), birds, amphibians and fishes [25, 34]. The latter included coelacanth, spotted gar and ray-finned teleosts such as the Antarctic icefish *Notothenia coriiceps*, cartilagenous fishes (sharks, rays and skates) and jawless fishes (lampreys, hagfish) in order to span all classes of extant vertebrates.

**Fig. 2:**
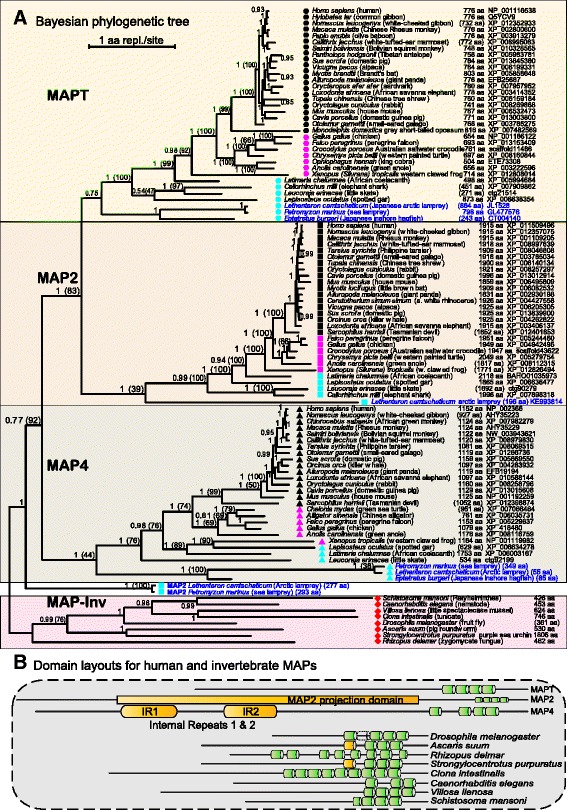
**a** Bayesian consensus phylogenetic tree of the MAPT/MAP2/MAP4 family. Putative homologous proteins of MAPT, MAP2 and MAP4 were retrieved from the NCBI-GenPept and UniProt databases and either completed or reconstructed *ab initio* by manual curation from BLAST and HMMER comparisons of genome assembly and coding transcript sequences. Full-length proteins representing the full species range for each vertebrate subfamily and a nonvertebrate outgroup were aligned (1947 aa from 102 species) and analyzed to consensus with ExaBayes on the Hanover supercomputer. Posterior probabilities and ML bootstrap percentage confidence values (in brackets) for the branching topology are shown at the nodes and branch lengths (SBL 63.9) are proportional to the amount of evolution along the horizontal scale (non-linear time). The branching topology was well supported and conformed to the known species divergence order identified by taxon symbols and descriptive labels. **b** Protein domain architectures (MTBD) representative of the vertebrate subfamilies were observed to contrast with various nonvertebrate homologs included in the phylogenetic analysis

Maximum likelihood analysis by RAxML, ExaML and MEGA of an alignment with 1949 sites in 102 species established the emergence of MAP4 from a metazoan ancestor and this was confirmed by the Bayesian consensus tree from ExaBayes with congruent topology and superior confidence values (Fig. 2). Worm, mollusc, insect and urchin MAPs exhibit similar domain architectures with shorter amino termini, while the tunicate *Ciona intestinalis* and fungus *Rhizopus delemar* are distinct (Fig. 2b). MAP4 was therefore considered to originate in the earliest vertebrates (hagfish and lampreys) and subsequent duplication of a more evolved common ancestor led to the formation of MAPT and MAP2 as sister genes. With this in mind, the strong evidence for MAPT full-length orthologs in hagfish and lamprey implied that MAP2 should be present in these same species; hence it is noteworthy that the lamprey branches for “short fragments” near the base of Fig. 2a were indeed recognized as MAP2 orthologs by the most significant matches of HMMSCAN to the pHMM digital template of MAP2. Bootstrap support at most bifurcations was highly significant in Maximum Likelihood, Neighbor-Joining and Bayesian posterior probability analyses. A more comprehensive dataset consisting of 2029 positions for 292 sequences was similarly analyzed by RAxML and ExaML (Additional file 1: Figure S1) to corroborate the results in greater detail, confirming the expected order of intermediate species with consistent branch lengths and, especially, the orderly separation of MAP4, MAP2 and MAPT in early vertebrates.

Taken together, our detailed reconstruction of the MAPT/MAP2/MAP4 superfamily of proteins from more than 300 sequences indicated that MAP2 and MAPT are encoded by sister genes that originated from a later common ancestor to MAP4. Furthermore, the data suggest that MAP4 emerged from a nonvertebrate ancestor with similar MTBD architecture.

### Identification and comparison of specificity determining positions in MTBDs

The order and timing of MAP gene duplications from phylogenetic analysis were consistent with the extended species distribution of many novel homologs described here. All this information validated the clades of confirmed orthologs for each gene into subalignments from which to build individual molecular profile hidden Markov models (pHMM) of each subfamily.

**Fig. 3:**
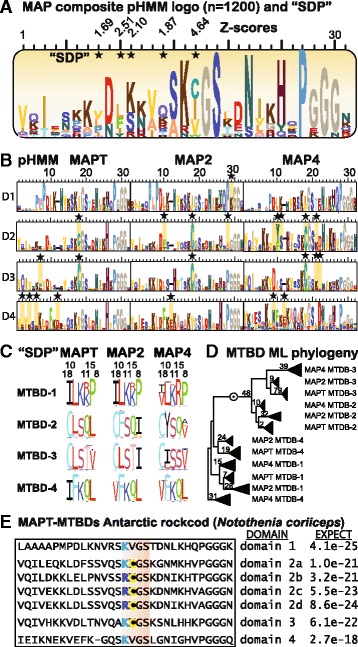
**a** pHMM sequence logo of the 4 microtubule binding domains in the 3 protein subfamilies MAPT, MAP2 and MAP4. More than 1200 individual MTBDs were aligned to build a pHMM and saved as sequence logo in scalar vector graphics format. The interpretation of amino acid distributions and column heights is summarized in Fig. 4 legend. Those sites characterized by Z-scores from SDPPRED as having distinct but conserved aa between the 4 different MTBDs in 3 paralogous subfamilies of a subclassified alignment of the 1200 domains, are shaded and starred as “specificity determining positions” responsible for functional divergence. **b** The 12 individual subfamily logos enable a direct comparison of all MTBD molecular profiles. The aa replacement of Ile/Val for Cys in the core tubulin binding motif “KCGS” of domains 2 and 3 was the most significant (Z-score 4.64 in A) specificity determining site (starred) and the deletion at position 12 in domains 1–3 differentiates these from domain 4. **c** SDP sequence logos of 5 sites in MTBD subalignments of 100+ proteins each with the highest Z-scores from SDP-PRED analysis. **d** Maximum likelihood analysis of the 12 MTBD subalignments of 100+ proteins each (using RAxML, WAG substitution model, 100 bootstrap pseudoalignments and gamma rates with alpha = 1.3). The point of separation of domains 1 and 4 from 2 and 3 was based on a midpoint root reflecting in the evolutionary relatedness of these domain pairs. Modest boostrap values were a consequence of the short, 33-aa sequence length and the triangle fans represent 100+ species orthologs for each MTBD category. **e** The influence of extreme cold adaptation on MAPT in the Antarctic rockcod *Notothenia coriiceps* was determined by reconstructing the transcript and deduced protein sequence from the corresponding genome assembly (gb:KL666590.1). The results showed 7 sequential MTBDs identified by their match score *E*-value to individual pHMMs (B above) with a 4-fold tandem duplication of MTBD 2 containing the typical central Cys preceded by an aa replacement from Lys to Arg

Our next aim was to define those sites likely to be responsible for the functional divergence between the paralogous families MAPT, MAP2 and MAP4 and within the MTBDs. This issue was addressed by examining the region containing the 4 well-conserved MTBDs in these subfamilies with programs such as SDPclust or SDPfox [35] that measure both the level of amino acid conservation at each site and the significance of any conserved changes (i.e. aa replacements) characteristic of paralogous divergence. A composite pHMM logo of 1200 MTBDs compiled in our studies (Fig. 3a) highlighted the core motif “KxGS” responsible for microtubule binding and the lesser prominence of the initial 6 aa of domains 2 and 3 implicated in paired helical filament (PHF) tangling of MAPT neuronal aggregates [10, 36]. A clustered alignment of the 4 individual MTBDs in the 3 subfamilies produced 12 defining logos that revealed isolated differences between these otherwise homologous domains (Fig. 3b) and scored those conserved sites that differed most significantly between the 12 classes. Figure 3a identified these divergent sites by Z-scores and stars while Fig. 3b highlighted the individual changes as gold-shaded residues. Noteworthy differences include the greater number of conserved prolines in domains 1, the conserved Cys in core domains 2 and 3 and the increase of acidic residues in domains 4. The replacement of I/V at positions 17–18 of MTBD repeats 1 and 4 with a prominent Cys residue in MTBD repeats 2 and 3 is a particularly significant change likely to be associated with differences in microtubule binding kinetics of the separate domains [36, 37]. The 5 highest scoring differences were summarized in Fig. 3c to delineate the conserved residues and/or changes at these informative positions 8,10,11,15,18 and a Maximum Likelihood tree of all 1200 MTBDs (Fig. 3d) supported the functionally divergent segregation of MTBDs 1 and 4 from 2 and 3 albeit with modest bootstrap support due to the shortness of the region analyzed (33 aa from 1200 MTBDs). The evolution of this domain may have introduced an alignment gap at position 12 in MTBDs 1, 2, 3 or insertion in MTBD 4 contributing to the divergence of the 3 subfamily members.

**Fig. 4:**
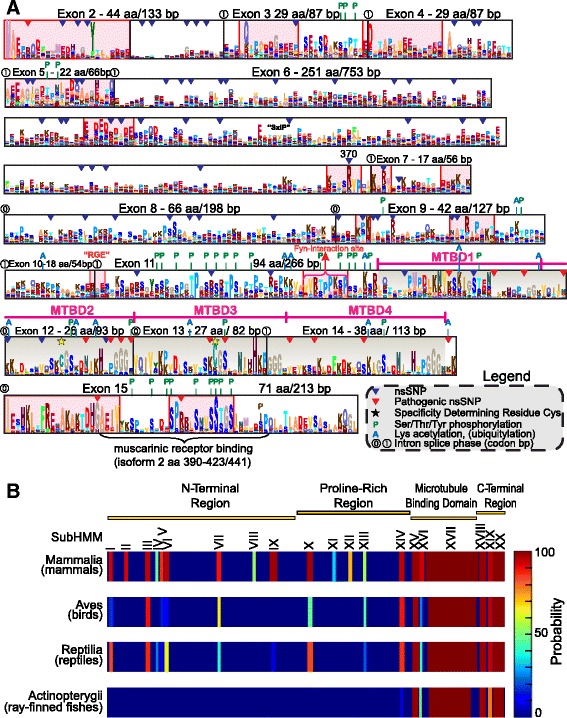
**a** Alignment pHMM logo for coding exons from full-length vertebrate MAPT homologs. The profile was reconstructed by SKYLIGN from a hidden Markov model based on a protein alignment of 117 orthologs validated by phylogenetic analysis. Exon numbers and lengths in amino acids and nucleotides are indicated with intron insertion phase numbers at exon junctures. Each site shows the relative proportion of 20 possible amino acids above background level (observed or hidden) and the total column height reflects the information content at each site, based on the overall conservation level imposed by functional constraint. Known MTBDs in exons 11–14 are grey shaded and sites inferred to possess some functional role (known or unknown) are shaded in red to emphasize their elevated column height conservation. The legend at the lower right summarizes other documented sequence features such as nonsynonymous population variants (inverted triangles), post-translational modifications (P-phosphorylation or A-acetylation) and specificity determining Cys residues (yellow stars). **b** Mammalian subHMM matches projected on a linear 776 aa human MAPT sequence. Each class is represented as a ribbon plot and each subHMM match is color-coded by the HHsearch match probability. The position of the match is indicated relative to the human sequence. The functional organization of tau is indicated on top similar to Fig. 1c. The single subHMMs are indicated in Roman numbers and the pHMM logos are displayed in Additional file 4: Figure S4

Comparative genomics can provide unique insight into the nature and extent of MAPT adaptation (i.e. evolutionary selection) to identify animal models of disease that might explain, for example, why non-human primates appear to be less susceptible to Alzheimer-like neurodegeneration despite having nearly identical primary sequences [38]. The search for other differences such as post-translational modifications [10] revealed a hyperphosphorylation state in hibernating animals [39] without pathological consequences. We investigated the MAPT primary structure in the Antarctic rockcod genome as an example of extreme cold adaptation because this fish is capable of efficient microtubule assembly at sub-freezing temperatures [40]. That study associated efficient microtubule assembly with amino acid replacement and glutamylation in fish tubulins. Interestingly, our pHMM models detected a modified architecture of the microtubule binding region consisting of 7 MTBDs including 4 tandem repeats of the second MTBD of MAPT with its characteristic Cys residue adjacent to mutated Lys → Arg residues (Fig. 4e). The predicted protein sequence is supported by similarity to one protein and 100 % coverage of the annotated genomic feature by RNAseq alignments including one sample with support for all annotated introns (NCBI GeneID: 104957078). Similar “anomalies” were also detected in a subset of other teleosts with 5 MTBD (UniProt: G3NJS9_GASAC, G3NJT3_GASAC, W5LPS2_ASTMX, W5ULM1_ICTPU), 6 MTBD (E7FH04_DANRE) or 7 MTBD (Q4S8L2_TETNG, A0A096MB04_POEFO). These internal duplications of exon 12 flanked by phase 0 introns and amino acid replacements would be expected to affect the oxidation-reduction capacity and positive charge in this region, critical as tubulin binding domains [37, 41, 42].

Our data indicate that MTBDs in the different subfamilies typically exhibit only small differences in their pHMMs, unlikely to alter microtubule interactions, but perhaps sufficient to assign them to their respective MAP subfamilies. The results suggest that the N-terminal projection region of MAPT may define the compartment-specific localization and tau-specific interactions.

### Identification and evolution of potential functional motifs in MAPT

The availability of full-length sequences from a broadly representative species distribution yielded an informative molecular fingerprint that highlighted regions of evolutionary constraint and conservation of amino acid properties from which to infer functional sites in the protein family. An alignment of 776 aa from 117 orthologs of full-length MAPT was used to build the corresponding pHMM with HMMBUILD and visualized as a sequence logo using SKYLIGN (Fig. 4a). Exons that encoded residues or regions with disproportionate aa conservation or elevated column height (shaded boxes) were inferred to be of functional importance, either for maintaining structural features or for providing interacting ligands to bind cellular structures (e.g. cytoskeleton or plasma membrane) or signaling molecules.

The 4 MTBDs (dark-shaded) encoded by MAPT exons 11–14 (Fig. 4a) highlighted the relative prominence of basic over acidic amino acids in the mechanistic role of these confirmed domains, in contrast to distinct composition and profiles in the poorly defined and sporadically conserved amino terminal region. Certain known characteristics of MAPT can be similarly identified in this logo format, such as the proline rich regions in exons 8–11 that contribute to structural turns in a secondary structure of 100 % coil predicted by PSIPRED, JPRED and other web-based algorithms (not shown). The phosphorylation-prone regions in exons 11 and 15 are relevant to the structural and functional changes common to neurodegenerative tauopathies [10, 43]. While these observations validated known features of this protein family, various other segments (pink-shaded) exhibited strong conservation of acidic residues in the amino terminus, a conserved RGE/KGE motif across the exon 10–11 splice site of one MAPT isoform, isolated regions in exons 2–5 and the extreme C-terminus, potentially conferring binding properties that affect other cytoskeletal or membrane components or MAPT conformation. We also observed a relative absence of hydrophobic, aromatic residues (Trp, Phe) that might contribute to α-helix formation and the presence of rare, conserved cysteines among the key central residues for microtubule binding of MTBDs 2 and 3, replaced by hydrophobic residues predicted for MTBDs 1 and 4. These and other structural features can be similarly extracted from the pHMM signatures of paralogous protein families MAP2 (Additional file 2: Figure S2) and MAP4 (Additional file 3: Figure S3). For example, a conserved Trp in exon 5, a characteristic KGE motif in exon 6, proline-rich exons 7 and 15 and a highly conserved C-terminus are distinctive features of MAP2. MAP4 harbors an amino terminus with particularly prominent conservation of diverse amino acids in exons 2–6, proline-rich exons 9 and 10, many basic Lys residues in the C-terminal half, very conspicuous MTBDs and prominent conservation at the extreme C-terminus.

While the C-terminal part containing the MTBDs is highly conserved between the MAPT/MAP2/MAP4 family, the N-terminal projection domain is likely to mediate the specific interactions of MAPT. A subHMM analysis of the pHMM of mammalian MAPTs identified conserved sequence motifs in the N-terminal region, subject to evolutionary selection and likely to be of functional importance. We identified 20 such motifs of which 12 were present in the N-terminal region (Fig. 4b and Additional file 4: Figure S4). By comparing these motifs with the pHMMs of birds, reptiles and ray-finned fishes we could follow their development during evolution. None of them were present in ray-finned fishes, while motifs I, III, VI, VII, and X were clearly evident also in birds or reptiles. Interestingly, motifs II, VIII, XI and XII were exclusively present in mammals, indicating that they represent functional regions peculiar to mammalian evolution.

### Display of exon structure, conservation and biophysical properties of MAPT onto one potential structural model

**Fig. 5:**
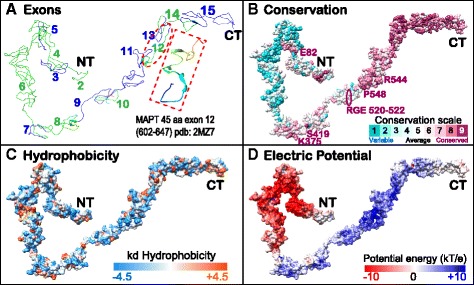
Display of exon structure, conservation and biophysical properties of MAPT on a potential structural model of human MAPTv6. Exon distribution (**a**) with red-boxed inset showing a MAPT fragment (602–647 in 776 aa isoform 6) that adopts a more stable helical structure when bound to tubulin [6], pbd:2MZ7; site-specific evolutionary conservation calculated by CONSURF (**b**); surface maps of hydrophobicity by CHIMERA (**c**); and surface electric potential from APBS (**d**) are shown. The predicted model with highest confidence score from I-Tasser was reconstructed by threading template fragments from the Protein Data Bank and *ab initio* modeling with consideration of steric constraints and low free-energy state. Note that MAPT is an intrinsically disordered protein without fixed constraints on 3D crystal or solution structure [18], so this model is intended mainly as a display platform for the protein physicochemical properties

The MAPT primary structure varies among orthologs in the multiple sequence alignments and corresponding pHMMs (Fig. 4) and these data provide a physicochemical basis for prediction of the secondary structure (not shown). However, it is becoming recognized that even a completely disordered 3D structure may adopt distinct regional conformations as a consequence of binding to molecular targets or cellular structures [5, 6, 18]. Despite the current lack of a static 3D crystal structure and difficulty in visualizing dynamic simulations from NMR solution structure or *in silico* modeling, we estimated one out of many possible 3D structures of full-length MAPT based on fragment template threading with steric and energy constraints using I-Tasser v4.1 [44]. The highest scoring model 1 described a fully coiled protein essentially without α-helices nor β-strands. We incorporated specific types of other information into this structure, such as exon distribution (Fig. 5a), site-specific residue conservation (Fig. 5b), hydrophobicity map (Fig. 5c) and electric charge distribution (Fig. 5d).

The exon layout follows the amino acid sequence of the displayed model in the I-TASSER structure file so that sites, motifs and domains of interest can be localized visually. The residue conservation pattern (Fig. 5b) also corresponded well to the pHMM model (Fig. 4). The amino terminal region included isolated patches like the extreme N-terminus, which may exert functional interactions with external binding targets or MAPT internal regions. The scattered distribution of hydrophobic residues contributes to the disordered structure [5] and precludes the formation of α-helices or transmembrane regions that are in fact non-existent in this model. Finally, the extreme polarity difference between negative amino and positive carboxy termini (Fig. 5d) is based on the primary amino acid composition of sites in the pHMM (Fig. 4) and provides a mechanism for large-scale folding interaction between these regions due to their opposing charges. Since disordered proteins may undergo promiscuous interactions depending on their momentary conformation, this model can serve to study diverse docking interactions with putative receptors wherein different regional, dynamic conformations may be adopted or simulated [4, 5, 14].

### Saitohin rediscovered

**Fig. 6:**
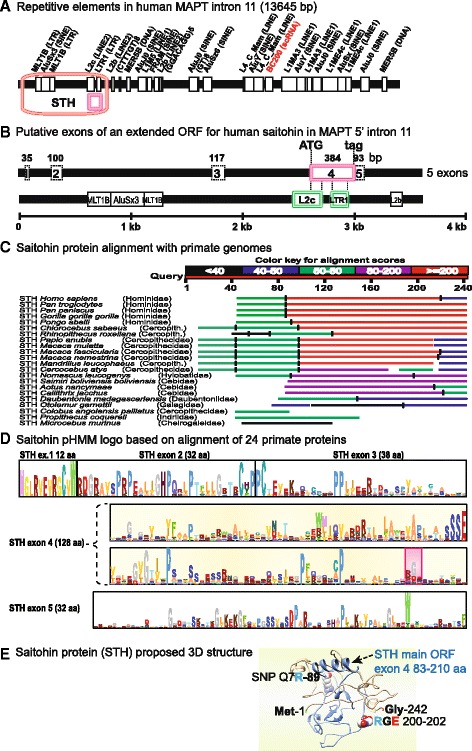
Saitohin (STH) gene locus in MAPT 5’intron 11. **a** Genomic repetitive element distribution in human MAPT intron 11 showing overlap of the saitohin main ORF with an L2c LINE2 element and an LTR1 long terminal repeat element predicted by REPEATMASKER (http://www.repeatmasker.org/). **b** An expanded view of the saitohin main ORF designated exon 4 here, overlapping with L2c and LTR1 elements within MAPT 5’intron 11, together with discontiguous, putative exons 1–3 and 5 encoding possible N- and C-terminal extensions. **c** Saitohin extended amino acid graphic alignment by TBLASTN for the 22 primate genomes listed. **d** A saitohin full-length pHMM reflects the relative conservation of individual amino acids as their probable frequency (letter size) while the information content or functional potential is reflected in total column height, exemplified by the conserved RGE motif marked in red highlight. This recognized exon 4 ORF is complete only for 7 Catarrhine apes, while N- or C-terminal ORF extensions with an upstream Met or downstream Stop codon have been observed for *Macaca fascicularis*, *Papio anubis* and *Pongo abelii*. Missense or nonsense mutations disrupt the main ORF in various species, although recent RNA Seq data may rectify the true genomic sequences to yield a longer translated protein in some cases (see text). **e** Saitohin protein 3D model (242 aa) predicted by I-TASSER with the confirmed single exon 4 ORF highlighted in blue, the disease-associated SNP “Q7R” at position 89 and an exposed RGE motif at positions 200–202

The single exon coding ORF within MAPT 5’intron 11 has been characterized by transcript expression and proteomic detection in certain hominoids [45, 46]. We reinvestigated its molecular evolution based on automated annotation of extended protein variants in other primates and abundant new RNA Seq data for transcript expression as part of the genome annotation pipeline of various species. The saitohin (STH) exon located in MAPT 5’intron 11 was found to overlap with REPEATMASKER predictions for 2 out of 400 genomic elements identified as L2c (LINE2) and LTR1 (long terminal repeat) (Fig. 6a). This suggested its possible origin from these elements in hominoids so we amplified this region (Fig. 6b) and mapped hypothetical extensions of the protein found in NCBI RefSeq protein annotations for STH from *Macaca fascicularis*, *Papio anubis* (amino terminus) and *Pongo abelii* (C-terminus) onto the human genomic sequence. Interestingly, the annotated protein extensions in these other species were based on RNA Seq coverage and mapped to “discontiguous” segments on the 5’ flank of MAPT intron 11, apparently describing a longer transcript encoded by multiple exons that were identified by location and size in Fig. 6b.

A TBLASTN alignment of 24 primate amino acid sequences (Fig. 6c) identified 7 great apes as the only species encoding the original exon 4 ORF for STH, whereas missense and nonsense mutations in other species precluded the manifestation of portions of this protein. More recent and extensive RNA Seq coverage may “correct” possible genomic sequencing “errors” for this exon 4 ORF and additional coding exons are now being included in alternate annotations to extend this peptide to an indeterminate number of other species. These data combined with our analysis of extended ORFs in certain species open the possibility that STH may be a multiexon encoded protein of variable length in different species (Fig. 6c). The longest extended ORF from *Macaca, Papio* and *Pongo* was used to reconstruct a full-length protein as a pHMM (Fig. 6d) and 3D model by I-TASSER (Fig. 6e) containing the single exon ORF originally described (blue structure). Taken together we were able to identify additional putative coding sequences for saitohin proteins in the MAPT gene of a limited number of primates and provided evidence that STH originates from two genomic elements, L2c and LTR1.

## Discussion

The conservation of MAP gene organization, chromosomal linkage and coherent phylogenetic analysis (Figs. 1 and 2 and Additional file 1: Figure S1) established the origin of MAP4, MAP2 and MAPT subfamilies in the earliest diverging vertebrates, separated from a nonvertebrate clade that included early chordates with distinct paralogous architectures. Reduced sequence identity and displaced or lost MAP exon splice sites in reptiles, amphibians and fish compared to mammals difficulted annotation in some cases but manual curation with custom pHMMs (Fig. 4) overcame various inconsistencies in the computer-predicted annotations. The discovery of MAPT, MAP2 and MAP4 in Agnatha (hagfish and lampreys) indicated that the gene duplication which led to the evolution of the MAPT gene occurred before the separation of jawless fishes (cyclostomes). Their confirmed presence in Chondrichthyes (chimeras, skates, sharks) sets their period of formation around 550+ million years ago.

Nonvertebrate MAPs form a separate outgroup clade, thus resolving nomenclature misnomers such as “tau” or “tau-like”. The phylogenetic order of gene duplication was rooted in ancestral metazoan homologs (from Platyhelminthes to marine chordates) with measurable separation from the vertebrate MAP4 clade. Subsequent ancestral duplication within the earliest vertebrates (hagfish, lamprey) created MAPT and MAP2 during the formation of jawless fish vertebrates. The zebrafish (*Danio rerio*) contains a duplicate MAPT gene, in addition to MAP4 and MAP2, possibly consequent to a teleost-specific genome duplication [47], analogous to putative segmental chromosome duplications producing multiple MAP copies in lamprey, Fig. 2 [48]. Indirect support from genetic linkage data associated MAPT and MAP2 with KANSL1 and KANSL1L, respectively, and myosin light chain MYL4, MYL1 and MYL3 may have been products of the same 3 paralogons during early vertebrate genome duplications [28, 29]. The genetic loci for MAP4 (3p21), MAP2 (2q34–q35) and MAPT (17q21.1) coincide with the proposed segmental chromosome duplications contained between 3p–17q and between HOX clusters in chromosomes 17-2–7–12 [30]. These analyses fill an important knowledge gap in MAP evolution because comparative genomics will be increasingly important to methodically define the genetic and protein structural variants relevant to physiological function, pathogenic mechanisms and disease processes.

Known and predicted structures (i.e. sites, motifs and domains) relevant to MAPT function were inferred from pHMM logos and complemented by data on conserved specificity determining positions (SDP) identified in protein subalignments and subHMMs. These novel, bioinformatic approaches were validated by statistical analysis of more than 100 vertebrate genomes by manual curation of full-length transcripts and proteins over a broad and uniform species distribution. The resulting pHMM column heights (Figs. 3 and 4, Additional file 2: Figure S2 and Additional file 3: Figure S3) and SDP Z-values (Fig. 3) demarcated known structural elements such as the MTBDs harbouring unique Cys residues [41], the potential for binding interactions of the amino terminus with the cell membrane [49, 50] and C-terminal binding region to muscarinic receptors [51]. Additional analysis with subHMMs revealed parts of the Fyn binding site [52], a possible EB1 binding "SIP" motif [53, 54] in MAPT at positions 255–257 (poorly conserved as "GIP" in mid-exon 6 of other species, Fig. 4) and other potentially functional motifs such as RGE/KGE [55, 56] that remain to be tested for novel cellular interactions. With both approaches (subHMM and SDP) we were able to identify conserved regions, which are candidates for functional interactions, and well-known longer regions like the MTBDs at the C-terminal part of MAPT. Our finding that additional conserved regions were present in mammalian MAPT compared to reptiles, birds and ray-finned fishes suggests that novel functions of tau evolved with increasing complexity of brain development.

The pHMM models were well-represented by full-length sequences from a broad range of species, hence they should not only reflect conserved, functionally important features but also predict potentially novel features such as externally interacting ligands for many putative interaction partners [9] and help to identify rare, disease-causing variants against a conserved primary sequence background. The alignments elaborated here can thus be compared to those from the ongoing Alzheimer’s genome sequencing project to search for suitable models and to determine ancestral alleles.

The fact that MAPT disorder and tangling are highly dependent on expression levels accentuates the importance of epigenetic regulation of gene expression [27], alternative exon splicing and post-translational protein modifications. The inclusion of complete molecular profiles was important to not exclude any structural feature that could be relevant to function or pathology. Comparisons between distant species can help to identify informative models [36]; for example, the modified MTBD architecture and aa composition of MAPT in Antarctic icefish (Fig. 3d) merits further comparative functional studies [40]; the saitohin ORF variation between monkeys and certain Catarrhine apes (Fig. 6) may be relevant to the more accentuated development of dementias in the latter group [45, 46]; the identification of key regulatory elements for transcription and alternative splicing in promoter and intronic regions and the impact of epigenetic changes [13] can also be studied by genomic bioinformatics analogous to the novel protein analyses presented here.

The 3D cartoon of MAPT presented here (Fig. 5) incorporated conservation data and protein properties such as hydrophobicity and electric charge distribution to provide clear evidence for regional differences that may help to understand MAPT interactions, internal folding properties and pathological aggregation. The charge distribution of amino acids in the amino versus carboxy terminal regions (Figs. 4 and 5) explains how the MTBD-containing C-terminus could bind directly to negatively charged phospholipids of internal plasma membranes whereas the MAPT amino-terminus may utilize intermediate ancillary proteins such as annexins to bind the plasma membrane [37, 49]. Hydrophobic residues show a dispersed distribution (Fig. 5c) as expected in an unstructured protein [3, 5] but closer examination in docking models might reveal their role in DNA and RNA interactions with phosphorylated MAPT [57].

The possible contribution of internal MAPT transcripts such as saitohin (STH) to MAPT regulation or function [45, 46] makes it of special interest in relation to tauopathies, other dementias and, more recently, schizophrenia. The fact that STH is known to bind MAPT [46] makes it of direct interest in relation to MAPT interactions, and the observation that complete single exon ORF STH may exist only in hominoids [45] is noteworthy because monkeys appear much less prone to tauopathies and clinical features of Alzheimer’s. The regulatory control of STH, chaperone and RNA expression requires further investigation in relation to MAPT transcription and subsequent processing. Since STH has no known homolog, it was of special interest to find that the main ORF coincided with and may have derived from two genomic repetitive elements. The issue of isoforms is even more complex and relevant to the expression of the individual MAPs, so our comprehensive, full-length annotation of all homologs will be instructive for detailed mapping of regional structures to specific functions and for interpreting differences in behavior among isoforms and species.

## Conclusions

The evolutionary origins of three paralogous members of the microtubule-associated protein family in vertebrates have been traced to the earliest vertebrates (Agnatha - hagfish and lamprey) during a period of whole genome/segmental chromosome duplications still evident in genetic linkage maps (Fig. 1). MAP4 derived from a nonvertebrate metazoan ancestor, while MAP2 and MAPT shared a more recent common ancestor with the same species distribution throughout the vertebrate subphylum (Fig. 2). The reconstruction of full-length proteins required de novo annotation of genomic sequences in early diverging vertebrates, to obviate isoform-specific differences and obtain representative profiles for phylogeny and modeling. Four conserved tubulin binding domains were consistently detected near the C-termini (Fig. 3), with internal domain duplications in Antarctic rockcod and certain other teleosts and markedly divergent N-termini in the most distant homologs. The phylogenetic classification of orthologs within each subfamily clade permitted correct alignment and building of individual profile HMM models (Fig. 4), yielding outlines of both conserved and variable sites and predictive probabilities for functionally important sites. These original models served to identify conserved domains with potential function, especially in the “uncharted” amino terminus. These features may be worthy of future investigation for possible roles in 3D protein structure determination or external binding and docking interactions (Fig. 5). The identification of associated genomic elements, such as linked KANSL and KANSL1L epigenetic enzymes, may be relevant to MAPT expression regulation, while repetitive elements identified in MAPT intron 11 may have given rise to a novel saitohin (STH) gene predicted to comprise multiple exons in additional hominoids (Fig. 6).

These original studies of MAP molecular evolution integrate much recent genomic data to provide a detailed roadmap for tracing the origins and structural variations of this important gene family. The resulting models of phylogenetic trees, pHMM, subHMM, SDP and 3D serve to predict new and known functionally relevant features of direct interest for interpreting the pathogenic properties of these proteins in neurodegenerative diseases and worthy of more focused investigation.

## Methods

Extensive and rigorous bioinformatic analysis of the microtubule associated protein (MAP) protein family was designed to extract functional information from sequence data of more than 100 vertebrate genomes. Specific aims were directed at the reconstruction of early diverging, full-length homologs by sequence search and assembly, phylogenetic analysis, pHMM model building with refinement as subHMMs and SDP, and the incorporation of evolutionary and physicochemical information into a static 3D model template.

Human MAPT, MAP2 and MAP4 reference genes were used with BLAST [58] and HMMER [59] programs to search, identify and retrieve homologous proteins from NCBI-nonredundant [60] and UniProtKB databases [61]. More than 100 vertebrate genomes were scrutinized to compile seed alignments of full-length proteins comprising all coding exons, using PROBCONS [62] and CLUSTALO [63]. Profile hidden Markov models (pHMM) were created using HMMER v3.1b2 [64] and partitioned into protein and nucleotide models of full-length transcripts, conserved domains and defined coding exons. These models were used to detect homologous sequences and were manually refined at exon borders. Subalignments of different species clades permitted the elaboration of specific training sets for more focused searches. These were further refined by PSI-BLAST and JACKHMMER searches to extend the ortholog/paralog list throughout vertebrates to the earliest diverging species. Genomic contig assemblies were retrieved from unannotated genomes [34] to encompass sequenced vertebrate and selected non-vertebrate genomes and identify all exons encoding complete transcripts.

Multiple sequence alignments were visually corrected and used to construct pHMMs and their corresponding logos, perform phylogenetic analyses, identify “specificity determining positions” (SDPclust, [35] and conduct protein sequence threading for *ab initio* 3D model predictions [44]. SubHMM analysis was based on the previously identified MAPT protein sequences and split into phylogenetic classes with at least eight species each (mammalian, aves, reptilian, actinopterygii). For each class a pHMM was built with HHSUITE [65]. The mammalian pHMM was split into subHMMs with a PYTHON adaption of the method proposed by [66]. The derived subHMMs were matched against the pHMMs of the other classes with HHSUITE.

Phylogenetic analyses were performed using Neighbor-Joining and Maximum Likelihood algorithms in MEGA v6.0 [67], RAxML v8.2 [68] and ExaML [69]. Subalignments were subjected to Bayesian implementations in PHYLOBAYES 3.1 [70], ExaBayes [71] and BEAST [72]. Parametric corrections were based on prior PROTEST v3 analysis [73] to select the WAG [74] or JTT [75] substitution model, 100–10,000 bootstrap pseudoalignments shown as node percentages, and 8–10 gamma rate categories with an estimated alpha (0.97–1.03) distribution. ML computational results using RAxML v8.2 and ExaML proved to be the most efficient, consistent and robust methods. Bayesian analysis performed with ExaBayes reached a consensus level with highest confidence posterior probabilities, while PHYLOBAYES 3.1 did not. Full-length proteins were used in all multiple sequence alignments except for partial sequences deduced from the earliest-diverging vertebrate genomes of hagfish, lamprey and elephant shark chimera. Metadata for the taxon list, alignment and phylogenetic tree (Fig. 2a) were deposited under TreeBase Study Accession (http://purl.org/phylo/treebase/phylows/study/TB2:S18990).

The pHMMs were visualized as sequence logos using SKYLIGN [76] to infer site-specific amino acid distribution as probabilities and those positions under functional constraint, based on letter and column heights, respectively. Note that pHMMs served to predict the probable sequence profile based on characters observed or absent from the source alignment. SDPfox [35] was used with clustered alignments of MTBDs from MAPT, MAP2 and MAP4 to identify specificity determining positions (SDP) by Z-score that were conserved among orthologous groups but different between paralogs and indicative of functional divergence. Structural 3D models were predicted by the I-TASSER server by threading the full-length MAPT sequence through matching template fragments in the Protein Data Bank [77] together with *ab initio* modeling based on steric, energy and charge constraints to achieve the highest confidence scores. The resulting best static model was displayed with UCSF CHIMERA [78] in one possible conformation of this otherwise disordered protein structure. Amino acid conservation values were obtained from the CONSURF server [79] and electric potential data were computed on the PDB2PQR-APBS server [80, 81].

### Availability of supporting data

Four supplementary Figures and Legends accompany the associated electronic file version. Accession codes for protein sequences are included in the figures and data are available at NCBI and UniprotKB.

## Additional files

Additional file 1: Figure S1. Amplified maximum likelihood phylogenetic tree of MAPT/MAP2/MAP4 family. The alignment was based on 1996 aa positions for 296 sequences, identified by name and actual protein length. Putative protein homologs of MAPT, MAP2 and MAP4 were retrieved from the NCBI-GenPept and UniProt databases and supplemented by manual annotation of genomic sequence data using pHMM models (nucleotide and protein) of all known exons and comparison with coding transcript sequences in the NCBI “nr, TSA and Ref. RNA” databases. Full-length proteins representing all vertebrate family and a nonvertebrate outgroup were aligned (1947 aa from 102 species) and analyzed by RAxML v8.2 with parameters (WAG substitution model, gamma rate correction with ML alpha, 100 bootstrap pseudoalignments, maximum likelihood computation value -113841). Bootstrap percentage confidence values for the branching topology are shown at the nodes and branch lengths proportional to the amount of evolution along the (non-linear) horizontal scale. The branching topology was well supported and conformed to the known species divergence order identified by taxon symbols and descriptive labels. (PDF 643 kb) Additional file 2: Figure S2. SKYLIGN sequence logo for coding exons from full-length vertebrate MAP2 homologs. The corresponding profile hidden Markov model was based on a protein alignment of 2167 aa in 102 orthologs validated by phylogenetic analysis (see Additional file 1: Figure S1). Coding exon numbers and lengths in amino acids and nucleotides are indicated with intron insertion phase numbers between exon blocks. Each site shows the relative proportion of 20 possible amino acids (observed or hidden) above background level and the total column height reflects 2 of 2 the information content at each site, inferred from over all conservation level due to functional constraint. The MAP2 projection domain in exons 9-11 is shaded grey and the 4 microtubule binding domains of 31–32 aa in exons 15–18 are shaded light brown to exemplify their homology with elevated site-specific conservation of known functional residues. (PDF 9571 kb) Additional file 3: Figure S3. SKYLIGN sequence logo for coding exons from full-length vertebrate MAP4 homologs. The corresponding profile hidden Markov model was based on a protein alignment of 1127 aa in 110 orthologs validated by phylogenetic analysis (see Additional file 1: Figure S1). Coding exon numbers and lengths in amino acids and nucleotides are indicated with intron insertion phase numbers between exon blocks. Alternatively spliced exons 3, 5, 11 and 12 have been omitted from this figure; compare with the longest possible isoform encoded by exons 2–23 (see Fig. 1). Each site shows the relative proportion of 20 possible amino acids (observed or hidden) above background level and the total column height reflects the information content at each site, inferred from over all conservation level due to functional constraint. The 4 microtubule binding domains of 31–32 aa in exons 15–18 are shaded light brown to exemplify their homology with elevated site-specific conservation of known functional residues. (PDF 5000 kb) Additional file 4: Figure S4. pHMM logos of subHMMs derived from mammalian pHMM logo by a Python adaption of a method proposed by Horan et al. 2010 [66]. The pHMM logos were built with SKYLIGN Web interface or by the underlying Perl scripts. The roman numbers refer to the subHMMs shown in Fig. 4b. (PDF 1 MB)

## Abbreviations

aa: amino acid
pHMM: profile hidden Markov model
MAP: microtubule associated protein
MTBD: microtubule binding domain
STH: saitohin
SDP: specificity determining positions
