# Supplementary figures and images for: An evolutionary roadmap to the microtubule-associated protein MAP Tau
Source: BMC Genomics. 2016 Mar 31;17:264. doi: 10.1186/s12864-016-2590-9 (PMC4815063; doi:10.1186/s12864-016-2590-9)

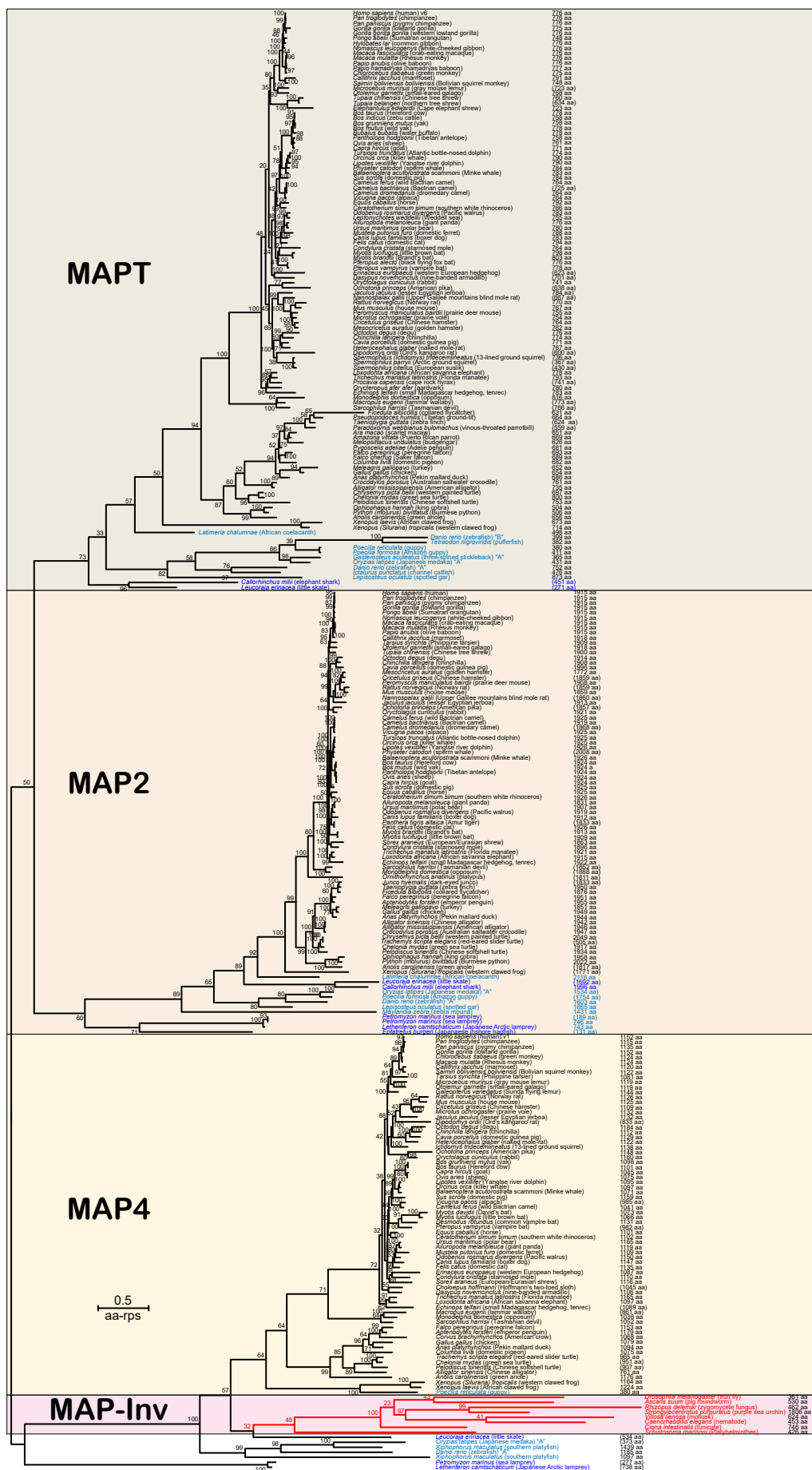

Supplement: Additional file 1: Figure S1. — Amplified maximum likelihood phylogenetic tree of MAPT/MAP2/MAP4 family. The alignment was based on 1996 aa positions for 296 sequences, identified by name and actual protein length. Putative protein homologs of MAPT, MAP2 and MAP4 were retrieved from the NCBI-GenPept and UniProt databases and supplemented by manual annotation of genomic sequence data using pHMM models (nucleotide and protein) of all known exons and comparison with coding transcript sequences in the NCBI “nr, TSA and Ref. RNA” databases. Full-length proteins representing all vertebrate family and a nonvertebrate outgroup were aligned (1947 aa from 102 species) and analyzed by RAxML v8.2 with parameters (WAG substitution model, gamma rate correction with ML alpha, 100 bootstrap pseudoalignments, maximum likelihood computation value -113841). Bootstrap percentage confidence values for the branching topology are shown at the nodes and branch lengths proportional to the amount of evolution along the (non-linear) horizontal scale. The branching topology was well supported and conformed to the known species divergence order identified by taxon symbols and descriptive labels. (PDF 643 kb) [file 12864_2016_2590_MOESM1_ESM.pdf]

## Mammalian MAPT subHMM logos

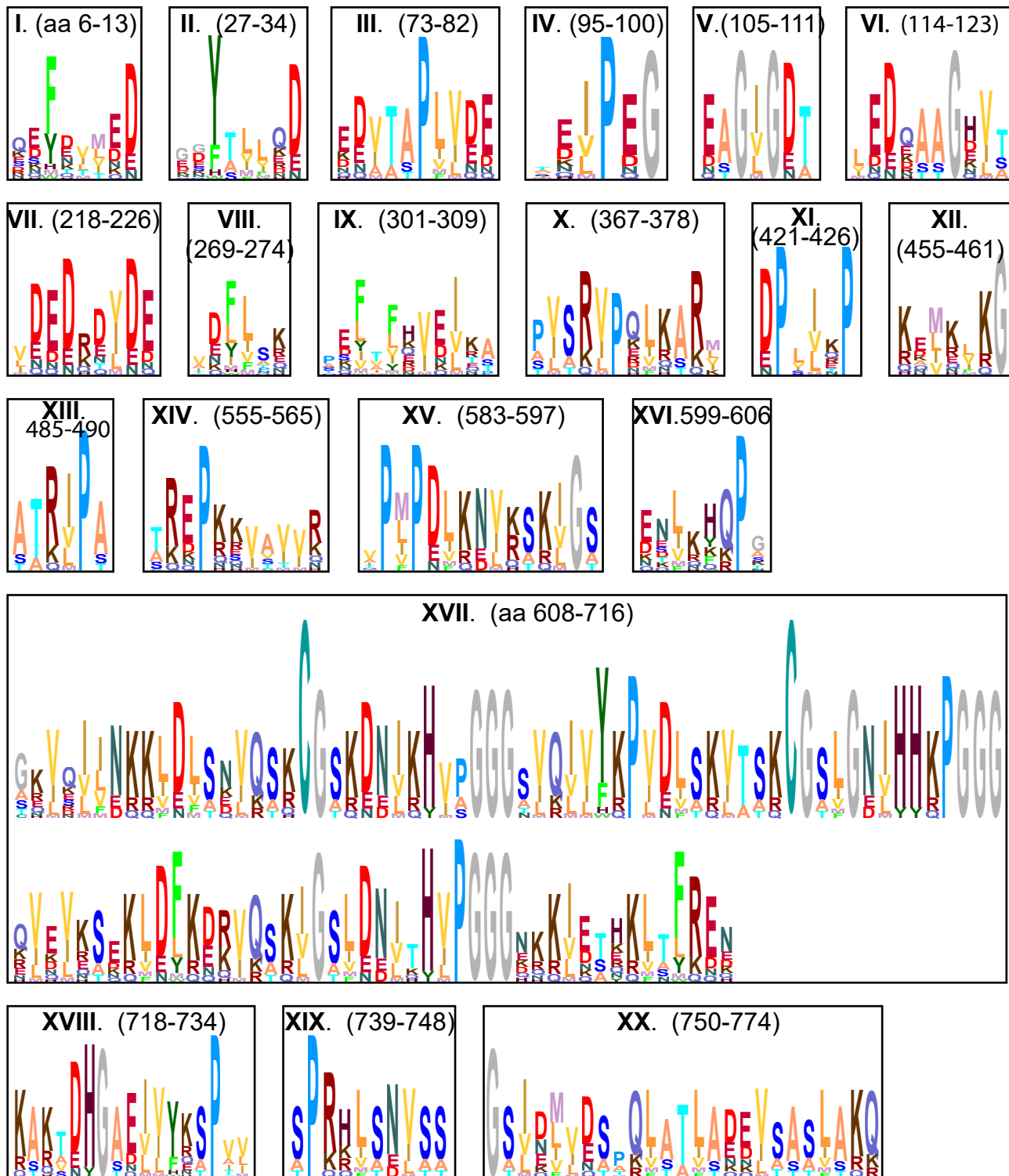

Supplement: Additional file 4: Figure S4. — pHMM logos of subHMMs derived from mammalian pHMM logo by a Python adaption of a method proposed by Horan et al. 2010 [66]. The pHMM logos were built with SKYLIGN Web interface or by the underlying Perl scripts. The roman numbers refer to the subHMMs shown in Fig. 4b. (PDF 1 MB) [file 12864_2016_2590_MOESM4_ESM.pdf]
