# Supplementary material for: An evolutionary roadmap to the microtubule-associated protein MAP Tau
Source: BMC Genomics. 2016 Mar 31;17:264. doi: 10.1186/s12864-016-2590-9 (PMC4815063; doi:10.1186/s12864-016-2590-9)
Supplement: Additional file 2: Figure S2. — SKYLIGN sequence logo for coding exons from full-length vertebrate MAP2 homologs. The corresponding profile hidden Markov model was based on a protein alignment of 2167 aa in 102 orthologs validated by phylogenetic analysis (see Additional file 1: Figure S1). Coding exon numbers and lengths in amino acids and nucleotides are indicated with intron insertion phase numbers between exon blocks. Each site shows the relative proportion of 20 possible amino acids (observed or hidden) above background level and the total column height reflects 2 of 2 the information content at each site, inferred from over all conservation level due to functional constraint. The MAP2 projection domain in exons 9-11 is shaded grey and the 4 microtubule binding domains of 31–32 aa in exons 15–18 are shaded light brown to exemplify their homology with elevated site-specific conservation of known functional residues. (PDF 9571 kb) [file 12864_2016_2590_MOESM2_ESM.pdf]

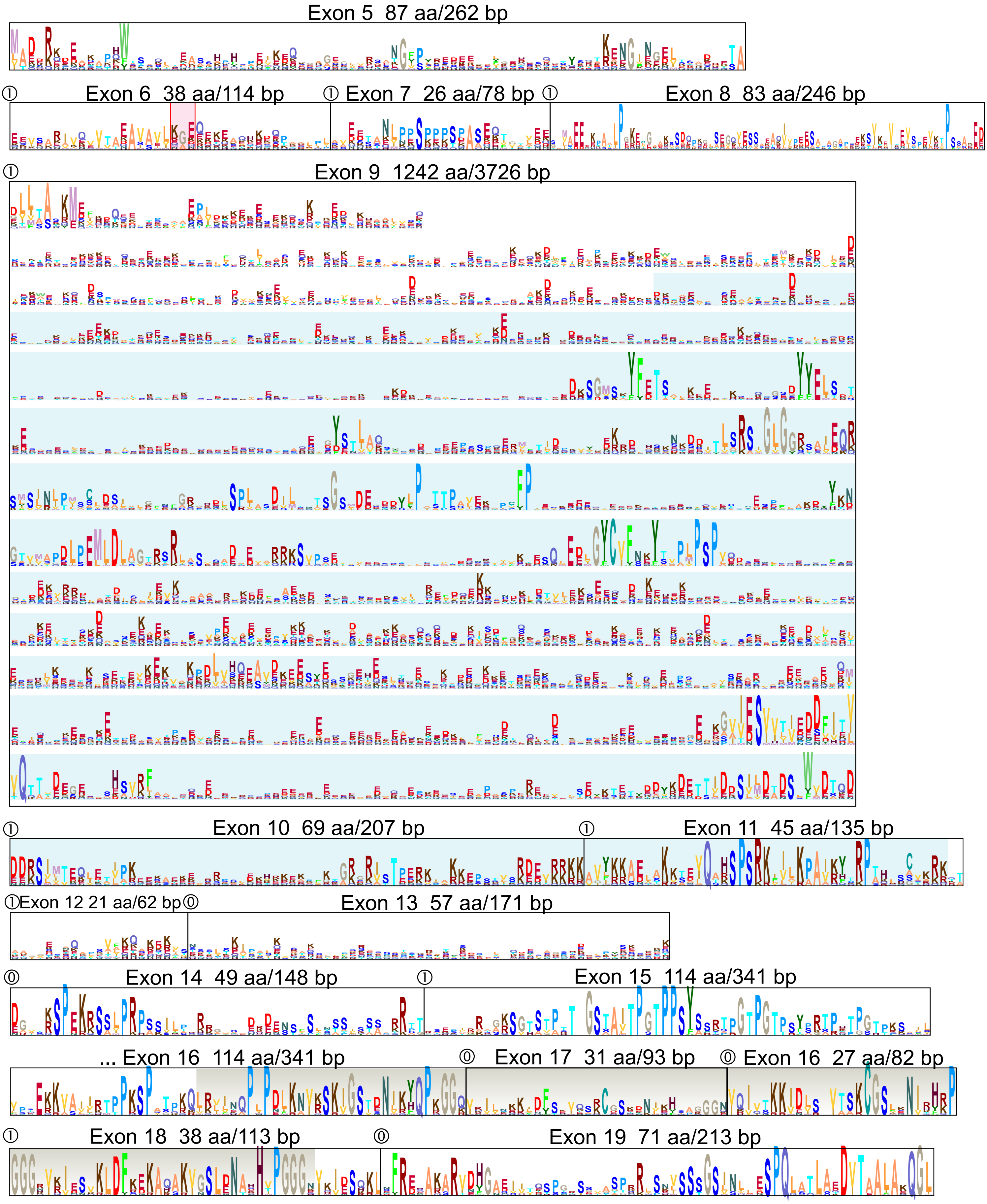

Exon 5 87 aa/262 bp
① Exon 6 38 aa/114 bp
① Exon 7 26 aa/78 bp
① Exon 8 83 aa/246 bp
① Exon 9 1242 aa/3726 bp
① Exon 10 69 aa/207 bp
① Exon 11 45 aa/135 bp
① Exon 12 21 aa/62 bp
⓪ Exon 13 57 aa/171 bp
⓪ Exon 14 49 aa/148 bp
① Exon 15 114 aa/341 bp
... Exon 16 114 aa/341 bp
⓪ Exon 17 31 aa/93 bp
⓪ Exon 16 27 aa/82 bp
① Exon 18 38 aa/113 bp
⓪ Exon 19 71 aa/213 bp
