# Supplementary material for: An evolutionary roadmap to the microtubule-associated protein MAP Tau
Source: BMC Genomics. 2016 Mar 31;17:264. doi: 10.1186/s12864-016-2590-9 (PMC4815063; doi:10.1186/s12864-016-2590-9)
Supplement: Additional file 3: Figure S3. — SKYLIGN sequence logo for coding exons from full-length vertebrate MAP4 homologs. The corresponding profile hidden Markov model was based on a protein alignment of 1127 aa in 110 orthologs validated by phylogenetic analysis (see Additional file 1: Figure S1). Coding exon numbers and lengths in amino acids and nucleotides are indicated with intron insertion phase numbers between exon blocks. Alternatively spliced exons 3, 5, 11 and 12 have been omitted from this figure; compare with the longest possible isoform encoded by exons 2–23 (see Fig. 1). Each site shows the relative proportion of 20 possible amino acids (observed or hidden) above background level and the total column height reflects the information content at each site, inferred from over all conservation level due to functional constraint. The 4 microtubule binding domains of 31–32 aa in exons 15–18 are shaded light brown to exemplify their homology with elevated site-specific conservation of known functional residues. (PDF 5000 kb) [file 12864_2016_2590_MOESM3_ESM.pdf]

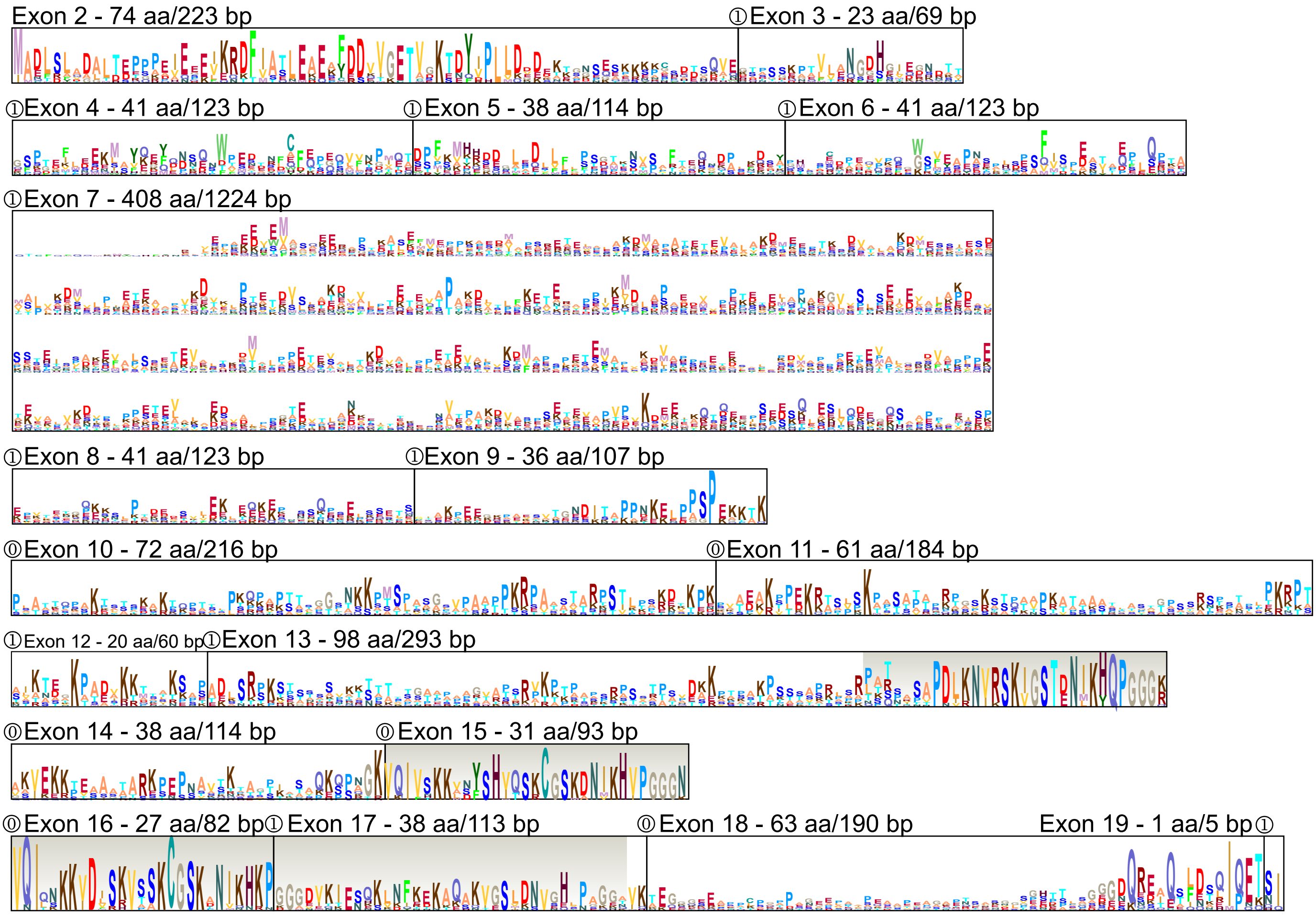
Exon 2 - 74 aa/223 bp
①Exon 3 - 23 aa/69 bp
①Exon 4 - 41 aa/123 bp
①Exon 5 - 38 aa/114 bp
①Exon 6 - 41 aa/123 bp
①Exon 7 - 408 aa/1224 bp
①Exon 8 - 41 aa/123 bp
①Exon 9 - 36 aa/107 bp
⓪Exon 10 - 72 aa/216 bp
⓪Exon 11 - 61 aa/184 bp
①Exon 12 - 20 aa/60 bp
①Exon 13 - 98 aa/293 bp
⓪Exon 14 - 38 aa/114 bp
⓪Exon 15 - 31 aa/93 bp
⓪Exon 16 - 27 aa/82 bp
①Exon 17 - 38 aa/113 bp
⓪Exon 18 - 63 aa/190 bp
Exon 19 - 1 aa/5 bp①
